# Alternative Insertion Site of Nexplanon: Description of a Case Report and Systematic Review of the Literature

**DOI:** 10.3390/jcm11113226

**Published:** 2022-06-06

**Authors:** Guglielmo Stabile, Carmelina Foti, Denise Mordeglia, Davide De Santo, Francesco Paolo Mangino, Antonio Simone Laganà, Giuseppe Ricci

**Affiliations:** 1 Institute for Maternal and Child Health, IRCCS Burlo Garofolo, 34100 Trieste, Italy; davide.desanto@burlo.trieste.it (D.D.S.); francesco.mangino@burlo.trieste.it (F.P.M.); giuseppe.ricci@burlo.trieste.it (G.R.); 2 Department of Medicine, Surgery and Health Sciences, University of Trieste, 34127 Trieste, Italy; carmelina.foti@burlo.trieste.it (C.F.); denise.mordeglia@burlo.trieste.it (D.M.); 3 Unit of Gynecologic Oncology, ARNAS “Civico—Di Cristina—Benfratelli”, Department of Health Promotion, Mother and Child Care, Internal Medicine and Medical Specialties (PROMISE), University of Palermo, 90127 Palermo, Italy; antoniosimone.lagana@unipa.it

**Keywords:** Nexplanon, subcutaneous device, etonogestrel implant, alternative insertion, implantation site, contraceptive implant

## Abstract

The etonogestrel (ENG) implant is among the most effective reversible contraceptives. It can be a good option for patients with different chronic diseases due to no clinically significant effects on lipid metabolism or liver function. Some limitations in the use of this type of device are represented by social and psychiatric disorders, where the easy accessibility of the device becomes a negative feature. In these patients several cases of self-removal or damage to the device have been reported. We report the successful insertion of the Nexplanon^®^ device into the scapular region in a young woman with a chronic psychiatric disorder. To verify the presence in the literature of other possible implantation sites, we performed a systematic review of the literature on Pubmed, Google scholar and Scopus from 2000 to 2021 using different combinations of the following terms: (Nexplanon), (contraceptive implant), (insertion). Two manuscripts with three cases were detected. Nexplanon^®^ was implanted in the upper back. In all cases, there were no complications during the insertions and the follow up demonstrated no side effects with contraceptive efficacy. Our report and review is a further confirmation that the scapular region can become a valid insertion site, maintaining good efficacy and safety of the subcutaneous device.

## 1. Introduction

Nexplanon^®^ is a progestin contraceptive implant placed under the skin on the inner side of the non-dominant upper arm for long-acting reversible contraception. It contains 68 mg of ENG implant that is slowly released for three years; it is not impacted by individual characteristics and genetic variants

The ENG implant is among the most effective reversible contraceptives available, with efficacy as good or better than the sterilization procedures, but without the risk of invasive surgery [1,2,3].

Implant contraceptives can be a good option for patients with chronic diseases because, beyond their need for highly effective contraception, there are no clinically significant effects on lipid metabolism or liver function. According to the criteria of eligibility of contraception [4,5], their use is contraindicated in women with serious conditions such as severe cirrhosis and other benign and malignant liver tumors, thromboembolic or ischemic heart disease, suspicious vaginal bleeding, breast cancer, systemic lupus erythematosus and anti-phospholipid positivity, or hypersensitivity to any component of the method [6]. Other possible limitations are represented by social and psychiatric disorders, where the easy accessibility of the device becomes a negative feature. In these patients, several cases of self-removal or damage (breaking or bending) of the device have been reported in the literature.

We report the successful insertion of the subcutaneous device in the scapular region in a young woman with chronic psychiatric disorder. To verify the presence in the literature of other possible implantation sites, we performed a systematic review of the literature on Pubmed, Google scholar and Scopus.

## 2. Case

A 16-year-old girl presented to our hospital, accompanied by her mother, for a menstrual disorder: she reported long periods of amenorrhea over the last years. The patient suffered from borderline personality disorder with marked affective instability and impulsivity with self-harm, which had caused multiple superficial cuts along the forearms bilaterally and on the right thigh. The patient was on chronic lithium therapy with poor control of psychiatric symptoms. Options were discussed and the mother, the legal administrator, refused the intrauterine device (IUD) because of the maidenhood status of the daughter, and the estrogen-progestogen pills due to the difficulty of ensuring daily intake. Additionally, the vaginal ring and transdermal contraceptive patch were inappropriate due to low adherence to treatment. For these reasons, the best option was the insertion of the subcutaneous device, Nexplanon^®^. Because of the patient’s psychiatric illness, the typical site at arm level was not recommended due to the high risk of self-removal or damage.

The risks and benefits of the subcutaneous device were explained to the mother and the patient, underlining that in the literature there are very few cases of alternative insertion site. They gave the consent for Nexplanon^®^ placement in a different unreachable body site.

**Figure 1:**
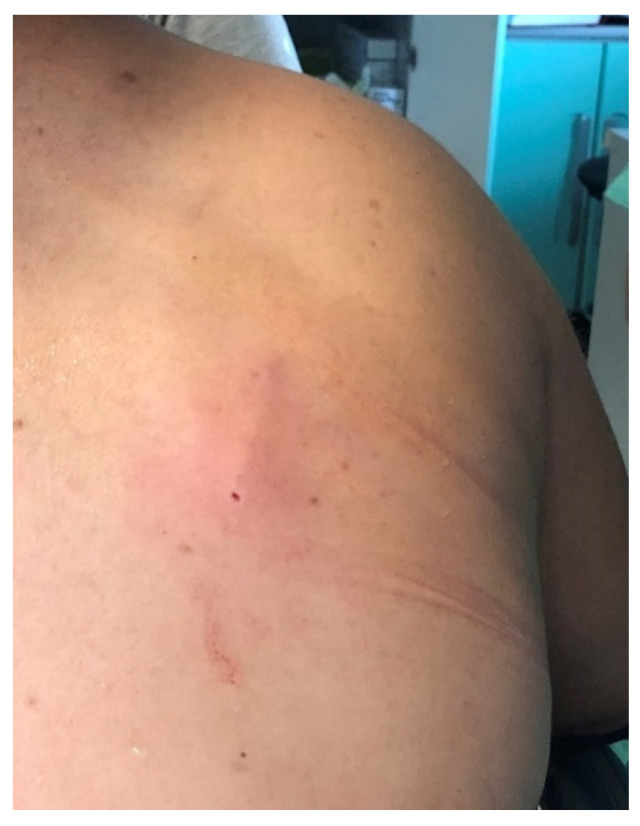
Nexplanon’s insertion site.

After skin disinfection and local anesthesia, Nexplanon^®^ was placed in the right scapular region without any complications (Figure 1).

At the follow-up visits (3 and 6 months after insertion), the patient reported normalization of the menstrual cycle without any side effects or discomfort related to the implant such as headaches, acne, pain, breast pain or depression. The implant remained superficial and palpable in the same position. However, she did not seem to pay attention to the implant and did not try to remove it.

## 3. Materials and Methods

This research was approved by our Institutional Review Board (RC 08/2020).

MEDLINE (PubMed), Google Scholar and Scopus databases were searched up to December 2021. The manuscripts considered were published from 2000 up to December 2021.

Only articles in English were included in the search. The research strategy adopted included different combinations of the following terms: (Nexplanon) AND (contraceptive implant) AND (insertion).

For the selection of the papers, we included articles that focused on cases of alternative sites of Nexplanon implantation. We examined in our review, the age and the history of patients, symptoms, the medical reason for a different Nexplanon^®^ implant, the procedure and the follow up.

We excluded from the review studies with different topics like aberrations from procedure for insertion and removal, and case complications such as infections, migrations, embolism, lipoatrophy and adverse reactions.

**Figure 2:**
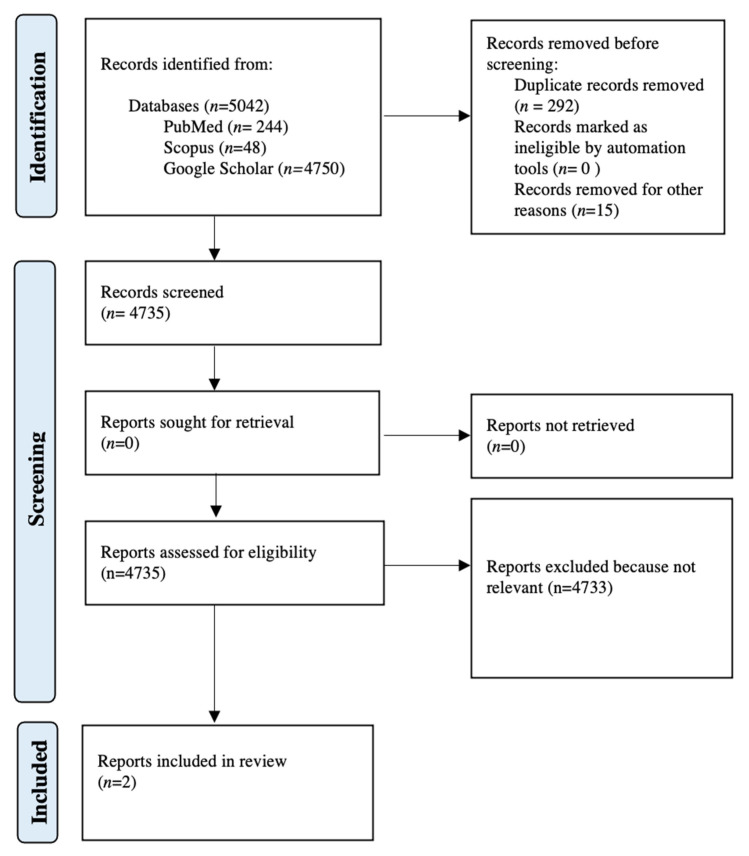
PRISMA flow diagram.

All studies identified were examined for year, citation, title, authors, abstract and their full texts. Duplicates were identified through manual screening performed by one researcher and then removed. PRISMA guidelines were followed. The PRISMA flow diagram of the selection process is provided in Figure 2. The systematic review was not submitted to Prospero as only a limited number of case reports were found in the literature. For the eligibility process, three authors independently screened the title and abstracts of all non-duplicated papers and excluded those not pertinent to the topic (G.S., C.F., D.M.). The same three authors independently reviewed the full text of papers that passed the first screening and identified those to be included in the review. Discrepancies were resolved by consensus. Two manuscripts were detected through the references of the works that had been identified with the research on PubMed, Scopus and Google Scholar [7].

Because this contraceptive method is cutting edge, the studies included are all case reports. For this reason, we present the data in a descriptive manner.

## 4. Results

We identified, through database searching, 5042 records (*n* = 244 from MEDLINE; *n* = 48 from Scopus; *n* = 4750 from Google Scholar). We removed duplicates (*n* = 292) and others written in non-English (*n* = 15). After screening and exclusion of all records on different topics, two manuscripts were detected as eligible in our review (Table 1).

They report three cases of young women (two twins and another girl) that suffer from chronic neurological diseases and psychotic symptoms, and that frequently self-inflict lacerations on their skin. For this reason, Nexplanon^®^ was implanted in the upper back. Only one had it placed under general anesthesia. There were no complications during the insertions and the follow up demonstrated no side effects with a good compliance and contraceptive efficacy.

## 5. Discussion

The Nexplanon^®^ implant is a 4 cm rod-shaped barium sulphate, containing 68 mg ENG.

Peak serum concentration (266 pg/mL) of ENG is achieved within 1 day after insertion, suppressing ovulation, which requires only 90 or more pg/mL [10]. Bioavailability of the ENG implant remains at nearly 100% over 2 years of use. After implant removal, serum ENG concentrations become undetectable within 1 week [11]. The contraceptive effect is obtained through two complementary mechanisms: the progestin inhibits the gonadotropin secretion and ovulation, and it causes changes in cervical mucus and tubal motility that are unfavorable to sperm migration [12].

To date, the common site of insertion is at the level of the medial face of the non-dominant arm with local anesthesia, 8 to 10 cm above the medial epicondyle of the non-dominant arm, 3–5 cm away from the sulcus between the biceps and triceps muscles that contains a large neurovascular bundle [13,14]. This anatomic area is easily accessible without important vessels and with low risk of device migration.

Complications are rare; they are reported in 0.3–1% of insertions and 0.2–1.75 of removals [15,16]. These include infection, hematoma formation, local irritation or rash, expulsion, and allergic reaction [17]. The implant may migrate a short distance (less than 2 cm) over time. There are two case reports of neurologic symptoms during ENG implant insertion in the literature: nerve injury to branches of the medial antebrachial cutaneous nerve during placement and neuropathy due to contact of the implant with the medial antebrachial cutaneous nerve [18,19]. In rare cases, intravascular insertion may cause migration of the implant to the pulmonary artery [20,21].

In the literature, there are very few cases of insertion in other regions (the median supraumbilical region and medial side of the thigh have been suggested) because of the presence of vulnerable nerves and vessels in the inner arm [8]. The scapular region is one of the less self-accessible areas and the anatomic site is distant from neurovascular structures, even in the case of accidental deep insertion, although it does not present a greater risk of migration.

We report the three cases described in the literature of insertion localized at the back and the results were good for compliance by the patient and contraceptive efficacy as demonstrated by the estrogen levels. In these cases, including the case from our institute, all patients shared similar diseases with psychiatric and neurological symptoms. In general, insertion of Nexplanon^®^ in other sites could be a good possibility in patients with social problems (e.g., drug addiction) or with skin and neuromuscular diseases of the upper limbs.

The strength of our study is the novelty and the presentation of a very rare case (only three others present in the literature) and the long period of time reviewed in the literature: we analysed the cases of alternative sites of implantation of contraceptive device from over the last 21 years. All the studies selected during the eligibility phase were further evaluated by manual comparison of populations, study settings and authors to avoid overlapping cases.

The limitation of our study is the retrospective nature of it and the main risk of bias is represented by the presence of all case reports among the papers selected, due to the rarity of this complication.

## 6. Conclusions

There are a significant number of psychiatric and social conditions that would benefit from the possibility of having an effective and safe contraceptive device, not easily accessible by the user. It becomes imperative to think of an alternative insertion site in order to meet all the needs of patients. As demonstrated by the previous cases described in the literature, our case report is a further confirmation that the scapular region can become a valid insertion site, because this anatomical region does not reduce the efficacy and safety of the subcutaneous device and does not lead to an increased risk of complications. Unfortunately, we do not know what the removal of the device will look like. For this reason, it is necessary to carry out a study that can systematically test this anatomical region as a possible alternative insertion site, evaluating the insertion, removal and associated complications compared to the common site.

## Figures and Tables

**Table 1:** Review of the literature.

Title	Authors	Age and Previous Pregranancies	Desease	Medical Reason of Nexplanon Different Insertion	Alternative Site	Anesthesia	Contraceptive Efficacy	Follow Up
Alternative insertion site in the scapular region for etonogestrel contraceptive implant (Nexplanon^®^) [8]	David Pragout MD, Francois Darrouzain Pharm.D PhD, Henri Marret MD PhD. “European Journal of Obstetrics & Gynecology and Reproductive Biology” 2018	23 years old nulliparous	Chronic psychotic illness that causes aggressive and self-harming behavior	Frequent self inflicted lacerations	Right lower scapular region	Local anesthesia	Good	3–6–18 months NO side effects, apart from amenorrhea
Novel location of Nexplanon^®^ placement in developmentally delayed twins: a case report [9]	Maura Quinlan MD MPH, Melissa Matulich MD. “Journal of Pediatric and Adolescent Gynecology” 2018	14 years old nulliparous	Global developmental delay, premenstrual behavior changes, hygiene issues with menses	Habitually pick at their skin	Upper back, parallel and 3 cm lateral to the spinous process at the level of the axilla	One twin: general anesthesia other twin: local anesthesia after oral anxiolytic	Good	No cyclic behavior issues have been noted and spotting has been tolerable

## Data Availability

All data were included in this manuscript.
